# L-Ferritin targets breast cancer stem cells and delivers therapeutic and imaging agents

**DOI:** 10.18632/oncotarget.10920

**Published:** 2016-07-29

**Authors:** Laura Conti, Stefania Lanzardo, Roberto Ruiu, Marta Cadenazzi, Federica Cavallo, Silvio Aime, Simonetta Geninatti Crich

**Affiliations:** ^1^ Department of Molecular Biotechnology and Health Sciences, Molecular Biotechnology Center, University of Turin, Turin, Italy

**Keywords:** ferritin, cancer stem cells, theranostic agents, magnetic resonance imaging, mammary tumors

## Abstract

A growing body of evidence suggests that cancer stem cells (CSC) have the unique biological properties necessary for tumor maintenance and spreading, and function as a reservoir for the relapse and metastatic evolution of the disease by virtue of their resistance to radio- and chemo-therapies. Thus, the efficacy of a therapeutic approach relies on its ability to effectively target and deplete CSC. In this study, we show that CSC-enriched tumorspheres from breast cancer cell lines display an increased L-Ferritin uptake capability compared to their monolayer counterparts as a consequence of the upregulation of the L-Ferritin receptor SCARA5. L-Ferritin internalization was exploited for the simultaneous delivery of Curcumin, a natural therapeutic molecule endowed with antineoplastic action, and the MRI contrast agent Gd-HPDO3A, both entrapped in the L-Ferritin cavity. This theranostic system was able to impair viability and self-renewal of tumorspheres *in vitro* and to induce the regression of established tumors in mice. In conclusion, here we show that Curcumin-loaded L-Ferritin has a strong therapeutic potential due to the specific targeting of CSC and the improved Curcumin bioavailability, opening up the possibility of its use in a clinical setting.

## INTRODUCTION

The occurrence of resistance to chemotherapeutic drugs, tumor recurrences and metastases formation show the difficulties in finding effective cancer treatments and raise the question whether current anti-cancer therapies target the right cancer cell population. There is a growing concern that the commonly used treatments might indeed miss a small population of tumor cells called “tumor-initiating cells” or “cancer stem cells” (CSC), composed of stem-like cells that play a critical role in cancer progression. Like normal stem cells, CSC have the capacity to self-renew and to give rise to a more differentiated progeny, and share common signalling pathways. According to the CSC theory, CSC can be: i) the source of all the tumor cells present in a malignant tumor and ii) responsible for tumor relapse and dissemination, being associated to the resistance to radiotherapy and to the conventional chemotherapeutic agents [1]. CSC have been identified in several human solid tumors and prospectively isolated through specific markers [2], although there is no general consensus on the best markers to identify these cells [3]. In human breast carcinoma, CSC have been identified for the first time by Al Hajj et al. [4] as a rare population of CD44^+^/CD24^−/low^ cells. Furthermore, CSC have the ability to survive and proliferate in anchorage-independent conditions giving rise to non-adherent spheres called tumorspheres that can be selectively cultured and expanded. This property was first described for neuronal progenitors [5] and then extended to progenitor cells of the mammary gland [6], to breast cell lines [7–9], and to human and murine breast carcinomas [10]. Given the central role of CSC in tumor progression, spreading and relapse, the cure for cancer might rely on CSC eradication. On the light of this therapeutic implication, several CSC-targeted approaches are being studied. These approaches range from indirect strategies, such as antiangiogenic therapies [1], to direct targeting, pursued through differentiation therapies, reversal of resistance mechanisms [1], and immunotherapy [11–13]. Another promising strategy consists in the identification of new and more specific biomarkers related to CSC status, which could serve as new targets, as we previously described [8, 9]. Moreover, these targets could be used for the development of new diagnostic tools able to provide an insight into the frequency of CSC within the tumor. Based on evidences obtained from both epidemiological and molecular studies, new insights are emerging linking the presence of excess iron and altered iron metabolism to cancer [14, 15]. Recently, Schonberg et al. [16] have demonstrated an enhanced iron scavenging ability in CSC of glioblastoma multiforme, due to a significant overexpression of the transferrin receptor (TfR) and a consequent increase in transferrin uptake, which is indicative of increased tumorigenicity. Therefore, targeting iron regulation within tumor-specific pathways could represent a potential approach for the development of new effective anti-cancer treatments.

In this study, we exploited the sphere-forming ability of CSC to selectively enrich the stem-like cell population present in a human (MDA-MB-231) and murine (TUBO) breast cancer cell lines, as described in [8], and for the first time we demonstrated that the uptake of L-Ferritin increased in CSC-enriched tumorspheres generated from both cell lines compared to their more differentiated counterparts. We surmise that this behaviour may be associated to their enhanced expression of the L-Ferritin receptor Scavenger Receptor Class A member 5 (SCARA5) that mediates Ferritin endocytosis [17]. Ferritin is the main iron storage protein and is composed of 24 subunits of heavy (H)- or light (L)-chain polypeptides that are present at different ratios in various organs to form a cage architecture of 12 nm in external diameter, with an inner cavity of 8 nm [18]. Once deprived of iron, this cavity can be used for the selective delivery of imaging and therapeutic agents to cells expressing Ferritin receptors [19]. The use of these nanotheranostic agents permits the non-invasive analysis of the pharmacokinetics and biodistribution of the nanomedicine formulation. Consequently, it is possible to monitor the efficacy of the therapy in real time and thereby to adapt the treatment regimens [20–22]. Several papers have explored the use of H-Ferritin to deliver selectively doxorubicin to cancer cells [23, 24]. In this contest, we have previously reported that the selective uptake of native horse spleen Ferritin and Apoferritin (composed by 85% and 15% L and H chains, respectively) loaded with MRI contrast agents and the anticancer drug Curcumin in the human breast cancer cell line MCF7 causes a significant reduction in cell proliferation *in vitro* [25]. Curcumin has been selected as therapeutic agent since it has been reported to exhibit anticancer activity *in vitro* and to be highly tolerated when administered to patients [26]. However, its poor water solubility and low bioavailability hampers its use as anti-cancer drug [27]. Therefore, loading Curcumin into Apoferritin can represent a solution for its delivery to cancer cells *in vivo*. Herein, we show that Apoferritin can be exploited for the simultaneous delivery of Gd-based MRI contrast agents and Curcumin for breast CSC targeting. Moreover, we show that administration of Curcumin-loaded Apoferritin leads to the regression of breast tumors *in vivo.* This approach could potentially enhance the responsiveness to current anticancer treatment regimens and might reduce the risk of relapse and dissemination of the disease.

## RESULTS AND DISCUSSION

### SCARA5 is upregulated in breast CSC

**Figure 1:**
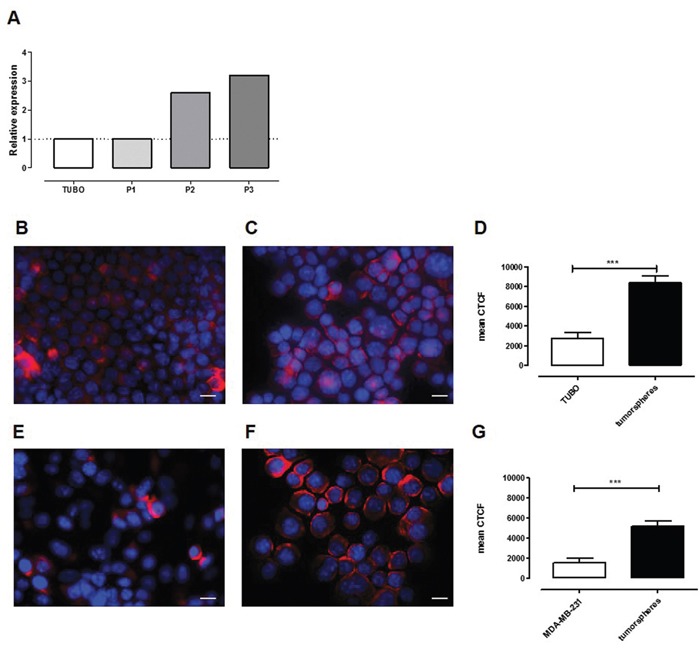
SCARA5 expression is upregulated in tumorspheres **A.** Relative transcript expression level of SCARA5 in TUBO cells and in three different tumorsphere passages. **B, E.** Representative images of TUBO and MDA-MB-231 cells or of **C, F.** their derived tumorspheres stained with an anti-SCARA5 mAb (red). Nuclei were counterstained with DAPI (blue). Scale bar, 20 μM. **D, G.** Graphs represent the mean ± SEM of the corrected total cell fluorescence (CTCF), calculated on at least 100 cells per sample as a quantification of SCARA5 expression in TUBO and MDA-MB-231 cells or in their derived tumorspheres.

A transcriptional analysis comparing the transcription profile of Her2^+^ murine TUBO cells cultured as monolayer with those of the first three *in vitro* passages of their derived CSC-enriched tumorspheres using MouseWG-6 v2.0 Illumina beadchips (GSE21451) [28] proved that SCARA5 is upregulated in tumorspheres (Figure 1A). SCARA5 protein expression increased from TUBO to tumorsphere-derived cells, as demonstrated by the representative images (panel B, C) and by the quantification of fluorescence intensity (panel D) reported in Figure 1. This enhanced expression is not restricted to TUBO-derived CSC, as it was also observed in tumorspheres derived from human triple negative breast cancer (TNBC) cell line MDA-MB-231 (Figure 1E-1G), further suggesting that SCARA5 may be a promising target of breast CSC.

### Breast CSC internalize more Ferritin than differentiated cancer cells

**Figure 2:**
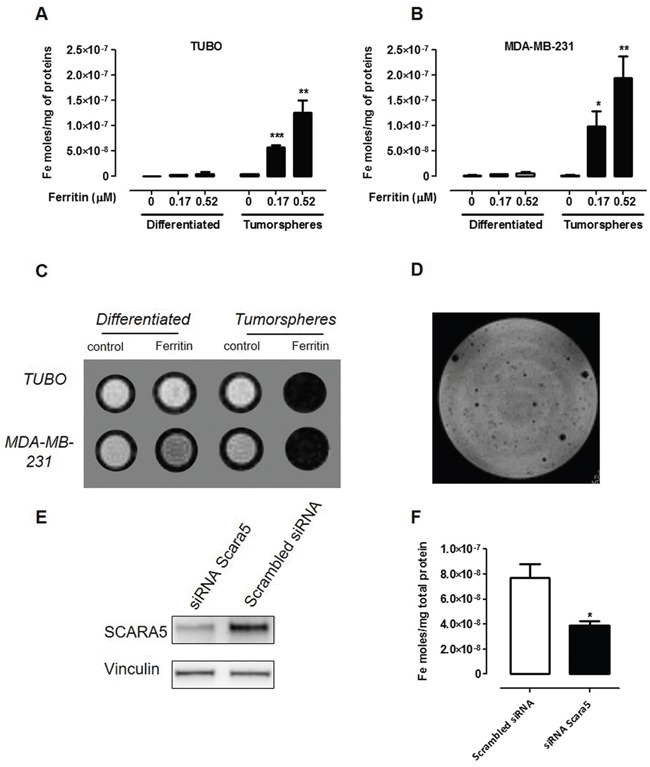
CSC display a higher Ferritin uptake than differentiated cells **A, B.** ICP-MS determination of the intracellular iron content of TUBO (A) and MDA-MB-231 (B) cells and their derived tumorspheres cultured for 24 hours with or without Ferritin 0.17 and 0.52 μM. Graphs show the mean ± SEM of internalized iron moles every mg of cell proteins from 3 independent experiments. **C.** A representative T_2_-weighted RARE MR image of an agar phantom containing TUBO and MDA-MB-231 cells (both differentiated and tumorspheres) incubated or not for 24 hours with L-Ferritin 0.52 μM. **D.** MRI of TUBO tumorspheres dispersed in agar. Each hypo-intense spot corresponds to the signal arising from one tumorsphere. **E, F.** TUBO derived tumorspheres were transfected with a siRNA to SCARA5 or a scrambled siRNA, and 48 hours after incubated with L-Ferritin for additional 24 hours. (E) Representative immunoblot of SCARA5 expression 48 hours after cell transfection. Vinculin expression was used as internal control. (F) Graph showing mean ± SEM of iron moles every mg of cell proteins, evaluated by ICP-MS, from 3 independent experiments. *p<0.05, Student's *t* test.

Since SCARA5 mediates L-Ferritin uptake [29], the first step of this study was the evaluation of the ability of MDA-MB-231 and TUBO tumorspheres to take up Ferritin from the medium compared to their corresponding more differentiated cells. For this purpose, horse spleen Ferritin, composed mostly of L-Ferritin chains and containing ca. 1000 iron atoms per protein, was used without any further modification. The experimental protocol consisted in the measurement of the amount of iron internalized by cells upon 24 hours of incubation in Ferritin-containing medium. The amount of internalized Ferritin was assessed by the ICP-MS determination of the intracellular iron content. The amount of internalized iron was significantly higher in tumorspheres than in differentiated cells, and increased with Ferritin concentration in both TUBO (Figure 2A) and MDA-MB-231 (Figure 2B) cell lines.

Ferritin contains a superparamagnetic ferrihydrite (5Fe_2_O_3_·9H_2_O) crystal that increases the transverse NMR relaxation rate (R_2_) of solvent water protons, causing a negative contrast in the corresponding MR images [30]. In order to assess whether native Ferritin can be exploited as a natural MRI probe for CSC detection, T_2_-weighted MR images were acquired following Ferritin incubation on both MDA-MB-231 and TUBO cells and their derived tumorspheres. Figure 2C shows that tumorspheres incubated with Ferritin displayed a markedly lower signal intensity when compared to untreated tumorspheres, while only small changes in signal intensity (SI) were observed in differentiated cells incubated in the absence or in the presence of Ferritin, respectively (Supplementary Material, Supplementary Table S1). Since spheroid diameter range is between 80 and 120 μm, TUBO-derived tumorspheres incubated 24 hours with 0.52 μM Ferritin were detectable as isolated spots after their dispersion in agar (Figure 2D). Altogether, these data show that Ferritin-based contrast agents may be exploited for the MRI detection of CSC. The specificity of the uptake was assessed by carrying out a competition study by incubating cells for 24 hours in the presence of an excess of native Apoferritin. In both MDA-MB-231- and TUBO-derived tumorspheres, the iron uptake measured by ICP-MS decreased of about 60% and 66%, respectively, confirming that iron uptake was mediated by Ferritin specific receptors. To ensure that the uptake of Ferritin by CSC is specifically mediated by SCARA5, we assessed the effect of SCARA5 silencing on the ability of cells to internalize Ferritin. TUBO-derived tumorspheres were incubated with a siRNA specific to SCARA5 or with a negative control scrambled siRNA, and the levels of SCARA5 protein were analyzed 24, 48 and 72 hours after transfection. SCARA5 transcript silencing (Supplementary Material, Supplementary Figure S1), led to a threefold decrease in SCARA5 protein level 48 hours after transfection, and this was maintained for the subsequent 24 hours (Figure 2E, Supplementary Figure S1). Hence, 48 hours after transfection, cells were incubated with 0.52 μM Ferritin for 24 hours. As shown in Figure 2F, the amount of iron internalized by cells treated with siRNA to SCARA5 was significantly lower than that internalized by control cells, thus confirming that SCARA5 mediates Ferritin uptake in breast CSC.

**Figure 3:**
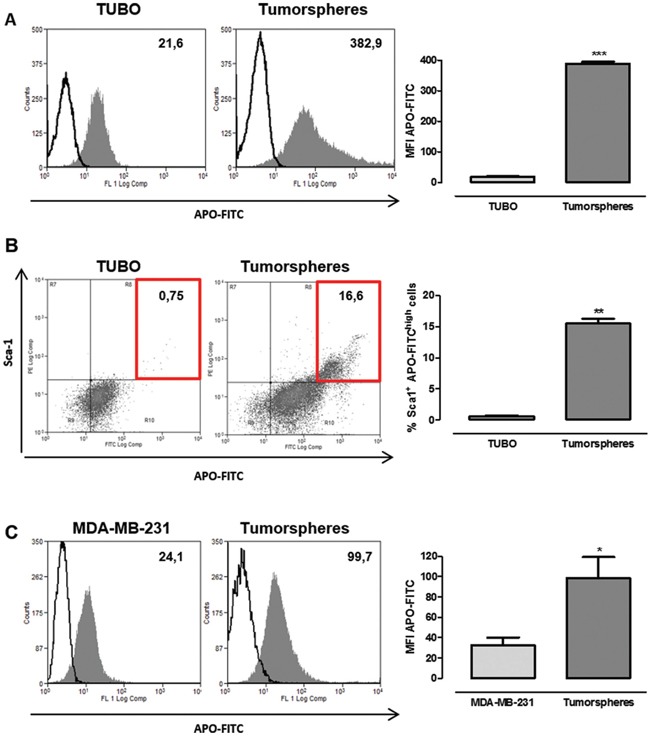
CSC display a higher APO-FITC uptake than differentiated cells FACS analysis of TUBO and MDA-MB-231 cells and their derived tumorspheres cultured with or without APO-FITC for 24 hours. **A, C.** Representative histograms of untreated (open histograms) or APO-FITC treated (gray histograms) TUBO (A) and MDA-MB-231 (C) cells and tumorspheres. Numbers show mean fluorescent intensity (MFI), the graphs show the mean ± SEM of APO-FITC MFI observed in cells and tumorspheres from 4 independent experiments. **B.** Representative dot plots of APO-FITC and Sca-1 expression in TUBO and tumorspheres. Numbers in quadrants show the percentage of APO-FITC^high^ Sca1^+^ cells (evidenced by the red boxes), the graph shows the mean ± SEM of the percentage of APO-FITC^high^ Sca-1^+^ cells in TUBO and tumorspheres from 4 independent experiments. *P < 0.05, **P < 0.01, ***P < 0.001; Student's *t* test.

In order to further confirm that breast tumorspheres take up more Ferritin than their differentiated counterpart, Apoferritin was labelled with FITC on its external surface (hereafter referred to as APO-FITC), and TUBO cells and their derived tumorspheres were incubated for 24 hours in the presence of APO-FITC. Flow cytometric analysis revealed that APO-FITC was internalized by both TUBO and tumorspheres, but its intake in tumorspheres was significantly higher, as demonstrated by their enhanced mean fluorescence intensity (MFI, Figure 3A) when compared to TUBO cells. Of note, all cells expressing the stem cell marker Sca-1 [8], only present in tumorspheres, displayed a higher APO-FITC uptake than the remaining cells (Figure 3B, Sca1^+^ APO-FITC^high^ cells in the red boxes), suggesting that Ferritin uptake is higher in CSC than in more differentiated cells. Similarly, a small amount of APO-FITC was internalized in MDA-MB-231 differentiated cells, while its internalization in their derived tumorspheres was significantly higher (Figure 3C).

### Uptake and intracellular distribution of rhodamine isothiocyanate labelled apoferritin (Apo-Rhod)

**Figure 4:**
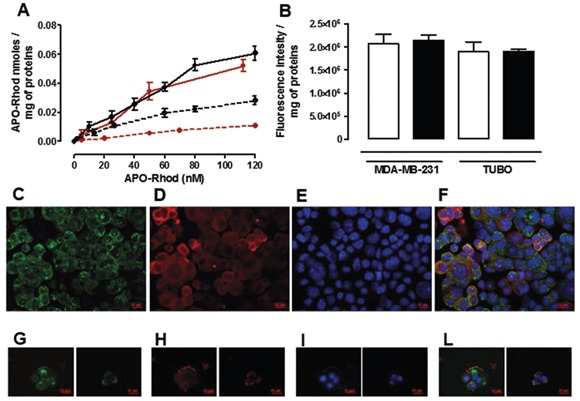
Uptake and intracellular distribution of Apo-Rhod **A.** Dose-response curve obtained by incubating different concentrations of APO-Rhod 24 hours with TUBO (red dotted line), MDA-MB-231 (black dotted line) and their derived tumorspheres (red and black continuous lines respectively). **B.** Apo-Rhod concentrations were determined by measuring fluorescence (ex/em 555/575 nm) on cytosolic extracts and were normalized to the cell proteins. Fluorescence intensity (au) of cytosolic extracts of TUBO and MDA-MB-231 derived tumorspheres, incubated in the absence (white bars) or in the presence of a 10 fold excess of H-Apoferritin. **C-L.** Representative images of TUBO derived tumorspheres incubated for 2 hours at 37°C with APO-Rhod (red, D and H) and stained with an anti-EEA1 antibody (green, C) or an anti-LAMP-1 antibody (green, G). Nuclei were counterstained with DAPI (blue, E and I). The co-localization between APO-Rhod and EEA1 or LAMP-1 is shown in the merged images of panels F and L, respectively. Scale bar, 10 μM.

In order to perform a dose-response study using low Apoferritin concentrations, thus evaluating its affinity to SCARA5 receptors, Apoferritin was conjugated on its external surface with Rhodamine isothiocyanate (hereafter referred to as APO-Rhod), and incubated 24 hours with TUBO, MDA-MB-231 and their derived tumorspheres. Figure 4A shows the amount of APO-Rhod taken up by cells, determined using a calibration curve performed with a Rhodamine standard solution. These results confirm that the amount of internalized APO-Rhod was significantly higher in tumorspheres than in differentiated cells even at nanomolar concentrations, which is an indication of the high affinity of Apoferritin for SCARA5 receptor.

In order to assess whether the presence of 15% of H-chains in the horse spleen Ferritin used in this study can mediate its uptake through H-Ferritin receptors, a further competition study was carried out by incubating both MDA-MB-231 and TUBO tumorspheres for 24 hours with APO-Rhod in the presence of a 10-fold excess of recombinant H-Ferritin [31, 32]. The H-Ferritin excess did not affect the amount of APO-Rhod internalized in both MDA-MB-231 and TUBO tumorspheres (Figure 4B) thus excluding the involvement of H-Ferritin receptors in the internalization of Horse spleen Apoferritin.

To explore the underlying internalization mechanism, APO-Rhod was incubated with TUBO-derived tumorspheres for fluorescence microscopy observation (Figure 4). Cells were then stained either for the early endosomal marker EEA-1 (panels C-F) or for the lysosomal marker LAMP-1 (panels G-L) to observe the process of APO-Rhod cellular trafficking. Two hours after incubation, APO-Rhod was located in the cytoplasm of most cells where it colocalized with early endosomes, as shown in panel F. LAMP-1 was distributed throughout the cytoplasm and the codistribution of APO-Rhod with LAMP-1 signal was circumscribed to the granular regions in the cytoplasm of some cells (Figure 4, panel L). These observations indicate that APO-Rhod was delivered to early endosomes after internalization. In the canonical receptor-mediated endocytosis pathway, early endosomes gradually mature to became late endosomes that then converge in lysosomes [33]. Most probably, APO-Rhod partly follows this path after internalization, since it colocalizes with the lysosomal marker LAMP-1.

### Uptake of apoferritin loaded with Gd-HPDO3A MRI contrast agent and Curcumin (Gd-APO-curcumin)

**Figure 5:**
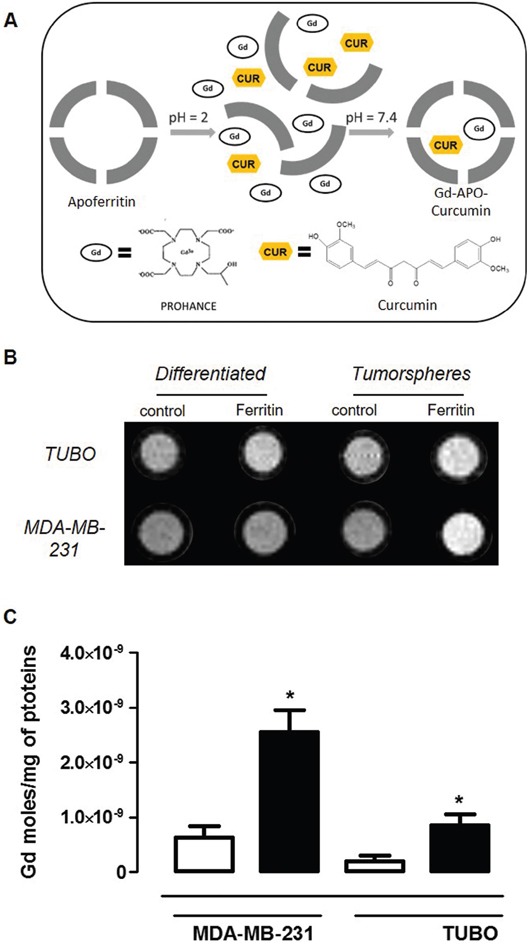
Gd-APO-Curcumin uptake is higher in CSC than in differentiated cells **A.** Schematic representation of Gd-APO-Curcumin preparation. **B.** A representative T_1_-weighted spin echo image of an agar phantom containing TUBO and MDA-MB-231 (both differentiated and tumorspheres) cells incubated or not for 24 hours with Gd-APO-Curcumin (2.7 μM in protein). **C.** Mean ± SEM of internalized Gd moles every mg of cell proteins from 3 independent experiments, measured by ICP-MS as in (B). *P < 0.05; Student's *t* test.

Despite its structural stability under physiological conditions, Ferritin displays a pH-dependent de-assembly, which can be exploited to load it with both therapeutic agents and imaging probes. Indeed, there are many examples where Ferritin nanoarchitecture was broken down in acidic environment and restored by retuning the pH to 7.4, after the entrapment of the desired solutes in its inner cavity [22, 34]. Using this procedure, Curcumin and the commercially available MRI contrast agent Gd-HPDO3A, neutral and safe even at high concentrations [35], were entrapped in the Apoferritin cavity (Figure 5A). Curcumin was selected as therapeutic agent since it has been reported to exhibit anti-oxidant, anti-inflammatory, anti-microbial and anti-cancer activity *in vitro* and in animal models of several diseases, such as acute hepatitis, acute ileitis, neuroinflammation, ischaemia and cancer [36]. Its anti-cancer activity is operated through modulation or inhibition of multiple molecular pathways [37]. Furthermore, during the past few years, a number of studies have suggested that Curcumin may have direct or indirect influence on CSC self-renewal pathways, including Wnt/b-catenin, sonic hedgehog, and Notch [38–40]. Another important property is that, unlike other known chemotherapeutic compounds, Curcumin does not cause any damage to normal cells [41]. Since SCARA5 is also expressed in the liver, the observation that Curcumin had no effect on normal rat hepatocytes [41] is essential to promote its use as a valid chemopreventive and chemotherapeutic agent for CSC using Apoferritin as a delivery platform. Furthermore, loading Curcumin into its protein cavity improves Curcumin bioavailability and stability under physiological conditions, maintaining its peculiar pharmacological properties [22]. Curcumin-loaded Apoferritin (APO-Curcumin) stability was assessed spectrophotometrically by measuring absorbance decrease at 430 nm. In the first 2 hours the absorbance showed a small decrease (15%), then it remained essentially constant during the entire experimental time (48 hours). Without Apoferritin, more than 90% of Curcumin decomposed rapidly (30 minutes) in buffer at neutral pH as already reported by Zebib et al. [42].

Using the protocol described above, the number of molecules that remained in Apoferritin inner cavity after the dissociation/reassociation procedure was 228 ± 48 and 9.6 ± 2 for Curcumin and Gd-HPDO3A, respectively. Gd-APO-Curcumin is stable for at least 48h as assessed by measuring absorbance at 430 nm [17]. The T_1_-weighted MRI image, recorded after 24 hours incubation in the presence of Gd-APO-Curcumin (2.7 μM), showed that the signal arising from labelled tumorspheres was clearly hyperintense compared to the untreated control tumorspheres for both cell lines (Figure 5B and Supplementary Materials, Supplementary Table S2). On the contrary, no significant signal enhancement was observed in Gd-APO-Curcumin-treated differentiated cells when compared to untreated cells. These observations were confirmed by the significantly higher amount of Gd taken-up by tumorspheres, measured by ICP-MS (Figure 5C). On the basis of the ICP-MS Gd measurements, it was possible to calculate the intracellular Gd, and consequently Curcumin, concentrations. In fact, since the Curcumin/Gd ratio in the Apoferritin preparation was 16, estimated intracellular Curcumin concentrations of 1400 and 460 μg/g were obtained for MDA-MB-231 and TUBO derived tumorspheres, respectively.

### APO-Curcumin induces cell death and reduces self-renewal of CSC

**Figure 6:**
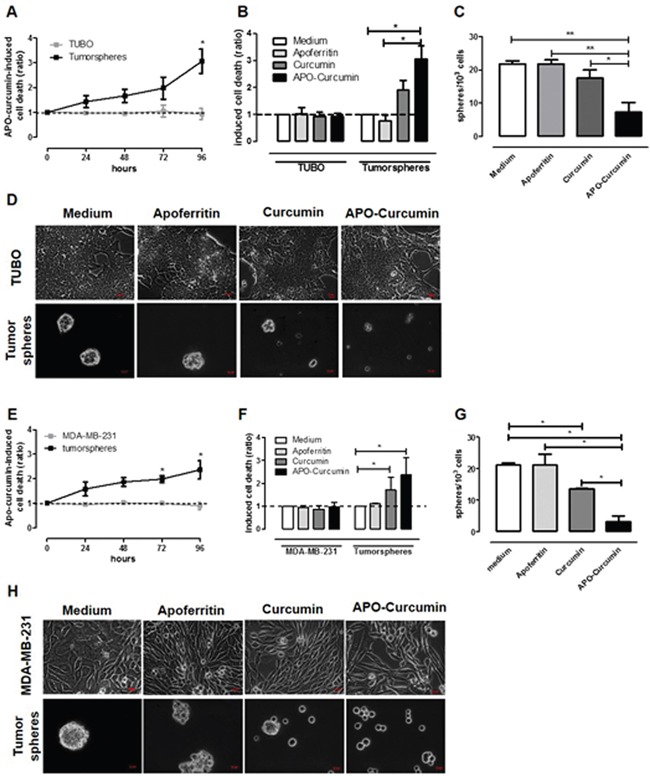
APO-Curcumin induces cell death and reduces self-renewal in tumorspheres TUBO **A-D.** and MDA-MB-231 **E-H.** cells were cultured as monolayer or tumorpsheres in presence of Apoferritin, APO-Curcumin or Curcumin. (A, E) After 24, 48, 72 or 96 hours, cells were stained with Annexin-V and PI and analyzed by FACS. The graphs show means ± SEM of the ratio of dead cells between APO-Curcumin-treated and untreated cells, calculated as described in M&M. (B, F) Graphs showing means ± SEM of the ratio of the percentage of dead cells present in samples treated for 96 hours with Apoferritin, APO-Curcumin or Curcumin in comparison to untreated samples. (C, G) The graphs show means ± SEM of the number of spheres generated every 10^3^ cells plated in the absence or presence of Apoferritin, APO-Curcumin or Curcumin. (D, H) Representative images of differentiated (upper panels) or tumorspheres (lower panels) cultured for 96 hours with the different treatments. Magnification 40X, scale bars 100 μm. All experiments were repeated at least 4 times. *P < 0.05; **P < 0.01; Student's *t* test.

In order to seek whether APO-Curcumin is able to induce inhibitory effects on CSC survival and self-renewal, TUBO cells and their derived tumorspheres where incubated in the presence or absence of APO-Curcumin for different time intervals, and cell death was evaluated by citofluorimetric analysis with Annexin V and propidium iodide staining. As shown in Figure 6A, APO-Curcumin did not induce cell death in TUBO cells, while progressively caused cell death in tumorspheres when compared to untreated cells. Of note, APO-Curcumin was more efficient than free Curcumin in inducing cell death in tumorspheres (Figure 6B). This enhanced effect was not due to any toxicity of the protein itself, since treatment with Apoferritin did not induce cell death in either TUBO or tumorspheres (Figure 6B), but likely to the ability of APO-Curcumin to enhance Curcumin uptake by CSC. APO-Curcumin and Curcumin affected not only tumorsphere viability, but also CSC self-renewal capacity as shown by a decreased ability of treated cells to re-generate tumorspheres, which on the contrary was not hindered by treatment with Apoferritin alone (Figure 6C). None of these compounds altered TUBO cell morphology (Figure 6D, upper panels), while tumorspheres cultured in presence of Curcumin and APO-Curcumin were smaller than those cultured in medium alone or with Apoferritin (Figure 6D, lower panels). The ability of APO-Curcumin to target CSC and decrease their survival and self-renewal is not restricted to the TUBO model, since similar results were obtained with MDA-MB-231 cells (Figure 6E-6H). While the increased effect of APO-Curcumin on tumorspheres compared to differentiated cells can be explained by tumorspheres’ increased expression of SCARA5 receptor, the reason why even free Curcumin (50 μM) affects tumorspheres but not differentiated cells is more speculative. It has been shown by others [38, 43] that Curcumin acts primarily on undifferentiated stem and progenitor cells rather than on more differentiated cells from mammary tissue and breast cancer cell lines. This effect appears to be mediated, at least in part, by the inhibition of intracellular signalling pathways that are upregulated in CSC [38]. It is therefore highly likely that a similar mechanism of action occurs also in our model.

### APO-Curcumin inhibits breast cancer growth *in vivo*

**Figure 7:**
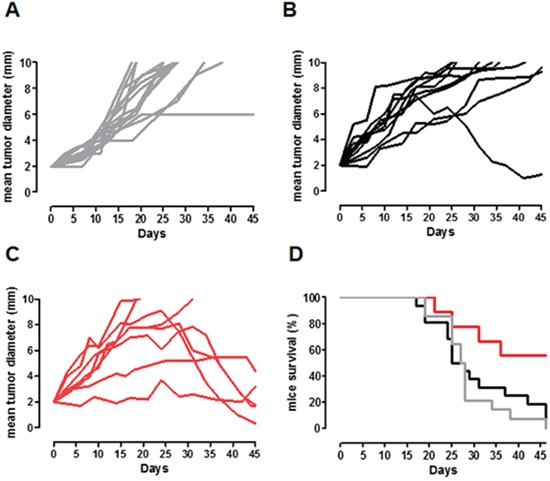
APO-Curcumin treatment delays CSC-induced tumor growth *in vivo* **A-C.** BALB/c mice were challenged s.c. with TUBO-derived tumorspheres and left untreated (**A**, gray lines) or treated i.v. with Apoferritin (**B**, 53 mg/Kg; black lines) or APO-Curcumin (**C**, 10 mg/Kg of Curcumin and 53 mg/Kg of protein; red lines) every 3 days, starting when their tumor reached 2 mm mean diameter. Each line depicts the growth of a single tumor. **D.** Kaplan-Meier survival curves. APO-Curcumin vs Apoferritin treated mice: *P*=0.0189; Apoferritin treated mice vs Untreated mice: *P* = 0.5; APO-Curcumin treated mice vs Untreated mice: *P*= 0.0054, Log-rank (Mantel-Cox) Test. Data were cumulated from two independent and concordant experiments.

To explore the efficacy of APO-Curcumin on established tumors *in vivo*, we set up a model that consists in the subcutaneous (s.c.) implantation of TUBO-derived tumorspheres into syngeneic BALB/c mice. After implantation, these cells maintain a prevalent CSC phenotype, as demonstrated by the presence of an higher level of both CD44^+^/CD24^−^ and Aldefluor^+^ expressing cells when compared to tumors generated by TUBO cells (Supplementary Material, Supplementary Figure S2). When tumors reached 2 mm mean diameter, mice were treated with APO-Curcumin or Apoferritin (10 mg/Kg of Curcumin and 53 mg/Kg of protein) intravenously (i.v.) every 3 days, or left untreated (Figure 7). The extremely low Curcumin water solubility prevents its direct i.v. administration without exploiting a nanoformulation/emulsion, therefore it was not possible to insert a Curcumin-treated group. Whereas virtually all tumors grew progressively in untreated or Apoferritin-treated mice (Figure 7A, 7B and Table 1), they did not progress or eventually regressed in about 60% of APO-Curcumin treated mice (Figure 7C and Table 1).

**Table 1:** Percentage of mice whose tumor regressed (responders) or grew (non responders) in the three groups

	APO-Curcumin	Apoferritin	Untreated
**Responders**	5/8 (62.5%)	1/10 (10%)	0/11 (0%)
**Non Responders**	3/8 (37.5%)	9/10 (90%)	11/11 (100%)

Data were cumulated from two independent and concordant experiments. APO-Curcumin vs Apoferritin treated mice: *P* = 0,043; Apoferritin treated mice vs Untreated mice: *P* = 0.47; APO-Curcumin treated mice vs Untreated mice: *P* = 0.0048, Chi square test.

Considering the animal survival, whereas all untreated mice died at the end of the experiment, about 60% of APO-Curcumin treated mice were still alive, compared to only 10% in the Apoferritin treated group (Figure 7D; *P*=0.018, Chi square test), suggesting that Curcumin-loaded Apoferritin may represent a promising tool for breast cancer treatment.

In order to verify that APO-Curcumin is not toxic to the liver, being SCARA 5 also expressed on hepatocytes, histological assessment of liver damage was carried out at the end point of the treatment. Periportal hepatocytes from both Apoferritin and APO-Curcumin treated animals did not show any injury but only a moderate degree of vacuolar degeneration, which is compatible with lysosomal compartment enlargement due to the endocytic uptake of the nanodevice (Supplementary Material, Supplementary Figure S3).

In conclusion, in this work it was shown that both human and mice breast CSC display an increased L-Ferritin uptake capability compared to their more differentiated counterparts. This is mediated at least in part by the upregulation of the L-Ferritin receptor SCARA5. These results open new horizons in the design of targeting strategies for the eradication of CSC, which are usually highly resistant to conventional chemo- and radiotherapy. Indeed, the increased L-Ferritin uptake can be exploited for the delivery of Curcumin to CSC, as the loading of Curcumin into L-Apoferritin does not cause any reduction of its affinity for the SCARA5 receptor. Of note, loading Curcumin into Apoferritin not only enables specific targeting to SCARA5-expressing cells, and thus to breast CSC, but also improves Curcumin bioavailability, opening up the possibility of *in vivo* treatments. Our *in vivo* results highlight the therapeutic potential of APO-Curcumin. However, to improve the clinical translatability of this approach, further experiments are needed in order to explore the synergistic effect of APO-Curcumin and classical chemotherapy. In this way, the elimination of CSC, exerted by APO-Curcumin, could be accompanied by the eradication of more differentiated tumor cells, leading to overcome tumor resistance and reduce recurrence. The relatively low sensitivity of MRI hampers the use of Gd-APO-Curcumin for the *in vivo* detection of CSC that represent a very low proportion of the tumor mass. However, this important objective might be achieved by developing more sensitive Apoferritin based PET or optical probes, which could provide new theranostic tools to improve breast patient stratification and monitoring of their response to therapy.

## MATERIALS AND METHODS

### Preparation of Gd-HPDO3A and Curcumin loaded Apoferritin (Gd-APO-Curcumin)

Gd-HPDO3A (ProHance) was kindly provided by Bracco Imaging S.p.A. Apoferritin, Ferritin (from horse spleen), Curcumin and all other chemicals were purchased from Sigma-Aldrich. H-Ferritin was kindly provided by Paola Turano (Center for Magnetic Resonance, University of Florence Italy) and prepared as described in [31, 32]. The loading of Curcumin and Gd-HPDO3A in the iron free Apoferritin cavity was carried out as described previously [22]. Briefly, the dissociation of Apoferritin into its subunits was done by lowering the pH of the 4.1 × 10^−6^ M protein solution (8 mL) to pH 2 using 1 M HCl and maintaining this low pH for about 15 minutes. Afterwards, 50 μL of a Curcumin solution in DMSO (200 mg/mL) and 2 mL 0.5 M Gd-HPDO3A were added to the Apoferritin solution. Then the pH was adjusted to 7.4 using 1 M NaOH. The resulting solution was stirred at room temperature for 2 hours, centrifuged, purified by gel filtration (Superdex G25 Column, Amersham) and dialysis. The solution was then concentrated using Vivaspin centrifugal concentrators (50 000 MWCO, Sigma-Aldrich). At the end of this process the concentrations of the protein and of Curcumin were measured by Bradford assay (using bovine serum albumin as a standard) and spectrophotometrically at 430 nm in ethanol, respectively. The final Gd concentration was determined by inductively coupled plasma mass spectrometry (ICP-MS) (Element-2; Thermo-Finnigan). Sample digestion was performed with 2 mL of concentrated HNO_3_ (70%) under microwave heating (Milestone MicroSYNTH Microwave Labstation).

### Cell lines

MDA-MB-231 were purchased from LGC Standards and grown in DMEM (Invitrogen Corp.) supplemented with 10% FBS (Sigma-Aldrich). TUBO cells [44] were generated from a spontaneous tumor of Her2/neu transgenic (BALB-neuT) mice [45] and were cultured in DMEM supplemented with 20% FBS. All cells were tested negative for mycoplasma by PCR assay and passaged in our laboratory for fewer than six months after their resuscitation. For tumorsphere generation, cells were detached and plated in ultra-low attachment flasks (Sigma-Aldrich) at 6 × 10^4^ viable cells/mL in mammosphere medium, as previously reported [8].

### Ferritin uptake experiments

MDA-MB-231, TUBO cells and their derived tumorspheres were incubated with increasing concentrations of Ferritin, Gd-APO-Curcumin and Apoferritin 24 hours post seeding. After 24 hours of incubation, cells were washed three times with ice-cold PBS and detached with trypsin/EDTA (Sigma-Aldrich). Then cells were lysed by sonication using an ultrasonic probe device (30% power). The Fe or Gd content in each cell line was determined by ICP-MS as described above. For MRI analysis (see below) cells were transferred into glass capillaries. The protein concentration was determined from cell lysates by the Bradford assay. The amounts of iron (measured by ICP-MS) internalized by cells were normalized to cell proteins concentration. 1 mg of proteins measured on cell lysate correspond to 3.1±0.3×10^6^ and 2.8±0.3×10^6^ MDA-MB-231 differentiated cells and tumorspheres, respectively, and to 2.9±0.4×10^6^ and 3.8±0.35×10^6^ TUBO differentiated cells and tumorspheres, respectively.

### MRI

All the MR images were acquired on a Bruker Avance 300 spectrometer (7 T) equipped with a Micro 2.5 microimaging probe (Bruker BioSpin). For *in vitro* determinations, glass capillaries containing 2 × 10^6^ cells were placed in an agar phantom and MRI was performed using a standard T_1_-weighted multislice spin-echo sequence (TR/TE/NEX = 250/3.3/8, FOV = 1.2 cm, NEX = number of excitations; FOV = field of view). T_2_-Weighted MRI images were obtained using a RARE sequence protocol (TR/TE/NEX = 5000/53/4; FOV= 1.2cm; MTX 128×128). The T_1_/T_2_ relaxation times were calculated using a standard saturation recovery spin echo. The image of tumorspheres dispersed in agar were obtained using a 3D FLASH gradient echo (TR/TE/NEX = 3500/18/2; FOV=1.14 cm; MTX 128×128×128).

### APO-FITC and APO-rhodamine preparation

2 mg/mL Apoferritin solution in 0.1 M sodium carbonate buffer at pH 9 was prepared. Fluorescein isothiocyanate (FITC, Sigma-Aldrich) or Rhodamine isothiocyanate (Rhod) were dissolved in anhydrous DMSO at 1 mg/mL. For each mg of protein, 50 μL of dye (FITC or Rhod) solution were added slowly (in 5 μL aliquots) while stirring the protein solution. When all the required amount of dye solution had been added, the reaction mixture was incubated in the dark for 16 hours at 4°C. Then NH_4_Cl was added to the solution to a final concentration of 50 mM, and it was incubated for other 2 hours at 4°C. The obtained Apoferritin solution was purified from non-entrapped dye with gel filtration using a G25 sephadex column, followed by dialysis. After purification, the solution was characterized in terms of protein concentration using the Bradford assay. The dye concentration was determined by measuring fluorescence (Horiba FluoroMax-4 spectrofluorometer), in Triton 0.1%, at 492/517 nm and 555/575 nm excitation/emission for FITC and Rhod, respectively.

### APO-Rhodamine uptake experiments

MDA-MB-231, TUBO cells and their derived tumorspheres were incubated with increasing concentrations of APO-Rhod 24 hours post seeding. After 24 hours of incubation, cells were washed three times with ice-cold PBS and detached with trypsin/EDTA. Then cells were lysed by sonication using an ultrasonic probe device (30% power) and rhodamine concentration in the cytosolic extracts was determined by measuring fluorescence as described in the previous paragraph. Competition study has been performed by comparing fluorescence intensity of TUBO and MDA-MB-231 tumorspheres incubated with APO-Rhod 25 nM (protein concentration) in the presence and in the absence of H-Ferritin 250 nM for 24 hours. Fluorescence intensity (expressed as arbitrary units) was normalized to the total protein cell content.

### Fluorescent microscopy

For SCARA5 detection, 3 × 10^5^ TUBO and MDA-MB-231 cells were plated on glass coverslips and left to adhere overnight at 37°C in a 5% CO_2_ incubator. 1 × 10^5^ tumorsphere-derived cells were cytospinned (Cytospin 4, ThermoScientific) to glass slides. Then, cells were fixed with 4% formaldehyde solution in PBS (Sigma-Aldrich) and stained with an anti-SCARA5 antibody (Thermo Scientific), as previously described [25]. To visualize the internalization of Apoferritin, 6 × 10^5^ disaggregated TUBO-derived tumorspheres were incubated for 2 hours at 37°C with APO-Rhod (0,47 μM in Rhod and 0,073 μM in Apoferritin). Cells were then washed twice with ice-cold PBS, cytospinned to glass slides, fixed in cooled methanol for 10 min and permeabilized in cooled acetone for 1 min. Slides were then stained with anti-EEA1 (Cell Signaling Technology) or with anti-LAMP-1 (Santa Cruz Biotechnology) antibodies and visualized with AlexaFluor488 goat anti-rabbit or goat anti-mouse AlexaFluor488 secondary antibodies (Invitrogen), respectively. Cells were visualized with an ApoTome fluorescence microscope (Zeiss). Photographs were taken by using a digital CCD camera and images were processed using the AxioVision 4.8 software. The mean corrected total cell fluorescence (CTCF) was calculated on at least 100 cells per sample, using the following equation: CTCF = Integrated Density of selected cell − (Area of selected cell × Mean fluorescence of background readings). All measurements were performed using the ImageJ software.

### Flow cytometric (FACS) analysis

Cells cultured as monolayers and their derived tumorspheres were incubated for different time intervals in the presence or absence of APO-Curcumin, free Curcumin, at a final Curcumin concentration of 50 μM, or Apoferritin (0.22 μM). Cells were then harvested and subsequently disaggregated using enzymatic and mechanical dissociation and washed in PBS supplemented with 0.2% BSA and 0.01% sodium azide (Sigma-Aldrich). Cells were then stained with the Annexin V apoptosis detection kit (eBioscience) according to the manufacturer's instructions. The amount of cell death induced by the treatments was calculated as ratio among the percentage of Annexin V^+^ and propidium iodide (PI)^+^ cells in treated samples compared to control cells.

To quantify APO-FITC uptake, cells were cultured with APO-FITC (1μM in FITC and 0.3 μM in APO) for 24 hours, then dissociated and stained with Alexa-Fluor647-conjugated anti-Sca-1 monoclonal antibody (Biolegend) as previously described [8]. All samples were analyzed using a CyAn ADP Flow Cytometer and the Summit 4.3 software (Beckman Coulter).

### CSC self-renewal assay

Tumorspheres generated from MDA-MB-231 and TUBO cells were dissociated after 5 days of culture and plated at the density of 6 × 10^4^ cells/mL in ultra low attachment six-well dishes in presence or not of APO-Curcumin, free Curcumin, at a final Curcumin concentration of 50 μM, or Apoferritin 0.22 μM. The total number of tumorspheres in each well was counted after 96 hours of culture and reported as number of tumorspheres generated per 10^3^ cells plated [8].

### Cell transfection

200 pmol of siRNA specific for SCARA5 (MSS291462) or negative control scrambled siRNA (Stealth RNAi Negative Control Hi-GC) were incubated with 10 μL Lipofectamin 2000 and diluted in Opti-MEM Reduced Serum Media (all from Thermo Fisher Scientific) according to the manifacturer's instructions. 1 × 10^6^ TUBO tumorsphere-derived cells suspended in 2 mL tumorsphere growth medium were incubated with the transfection mix and harvested after 24, 48 or 72 hours and used for further experiments.

### Western blotting

Cells were lysed with cell lysis buffer (0,5% NP-40, 150 mM NaCl, 50 mM Tris-HCl, 0,25 mM EDTA, 1 mM DTT, 1 mM Na_3_VO_4_ and 1:2000 protease inhibitor cocktail (all from Sigma-Aldrich)) as in [46]. 40 μg total proteins were resolved by SDS-PAGE and electroblotted onto PVDF membranes. After blocking with non-fat dry milk diluted in Tris-Buffered Saline – Tween (TTBS) 0,05%, membranes were probed with rabbit anti-SCARA5 (Santa Cruz Biotechnology) or mouse anti-vinculin (Santa Cruz Biotechnology) antibodies followed by HRP-conjugated anti-rabbit IgG or anti-mouse IgG (all from Sigma-Aldrich) and visualized with the ECL Western blotting substrate (Thermo Fisher Scientific), using a ChemiDoc Touch Imaging System (Biorad).

### *In vivo* experiments

BALB/c mice (Charles River Laboratories) were maintained at the Molecular Biotechnology Center, University of Turin, and treated in accordance with University Ethical Committee and European guidelines under Directive 2010/63. Mice were subcutaneously (s.c.) challenged with 1 × 10^4^ tumorsphere-derived cells (TUBO) and treated intravenously (i.v.) with APO-Curcumin (10 mg/Kg of Curcumin dose, corresponding to a protein dose of 53 mg/Kg) or Apoferritin (at the same protein dose) every three days, starting when they have developed a 2 mm mean diameter tumor, or left untreated. Tumor growth was monitored twice a week with a caliper and reported as the mean of two perpendicular diameters. When tumors reached 10 mm mean diameter, mice were euthanized for ethical reasons.

## SUPPLEMENTARY MATERIAL FIGURES AND TABLES


