# Supplementary material for: L-Ferritin targets breast cancer stem cells and delivers therapeutic and imaging agents
Source: Oncotarget. 2016 Jul 29;7(41):66713–27. doi: 10.18632/oncotarget.10920 (PMC5341832; doi:10.18632/oncotarget.10920)
Supplement: Supplementary file 1 [file oncotarget-07-66713-s001.pdf]

L-Ferritin targets breast cancer stem cells and delivers therapeutic and imaging agents

SUPPLEMENTARY MATERIALS AND METHODS

FACS analysis of CSC in tumors

Female BALB/c mice (Charles River Laboratories) were s.c. challenged with 1 × 10^4 tumorsphere-derived cells (TUBO) or with 1 × 10^5 TUBO cells, and tumor growth was monitored twice a week with a caliper. When tumors reached 10 mm mean diameter, mice were euthanized and tumors were explanted, finely minced with scissors and then digested by incubation with 1 mg/ml collagenase IV (Sigma Aldrich) in RPMI-1640 (Life Technologies) at 37°C for 1 hour in an orbital shaker. After washing in PBS supplemented with 2% FBS (Sigma-Aldrich), the cell suspension was incubated in an erylise buffer (155mM NH_4Cl, 15.8mM Na_2CO_3, 1mM EDTA, pH 7.3) for 10 minutes at room temerature. After washing in RPMI-1640 supplemented with 10% FBS, the cell suspension was passed through a 40 µm pore cell strainer, centrifuged at 1400 rpm for 10 minutes, treated with Fc receptor blocker (anti-CD16/CD32, BD Bioscences) and stained with PE-conjugated anti-CD44 and PE-Cy7-conjugated anti-CD24 antibodies (Biolegend) or with the Aldefluor kit (Stem Cell Technologies) according to the manufacturer's instructions. All samples were analyzed using a CyAn ADP Flow Cytometer and the Summit 4.3 software (Beckman Coulter).

Histopathological assesment

Liver fragments of 0.5 × 0.3 × 0.2 cm were fixed overnight in 4 % buffered formaldehyde solution at 4°C and processed by standard methods. Some 5 µm dewaxed sections stained with hematoxylin and eosin were used to evaluate the degree of damage.

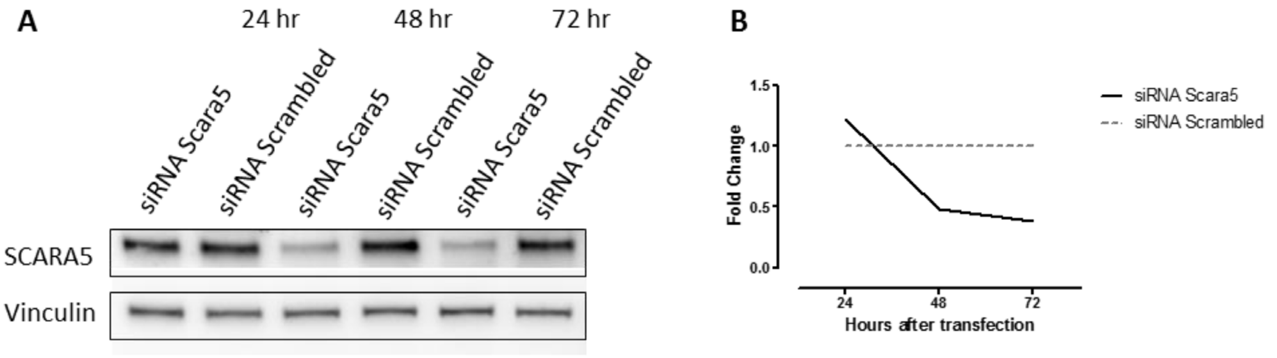

Supplementary Figure S1: Kinetics of SCARA5 silencing in tumorspheres. A. Immunoblot showing SCARA5 expression in TUBO-derived tumorspheres harvested 24, 48 or 72 hours after transfection with a siRNA specific for SCARA5 or with a negative control Scrambled siRNA. Vinculin expression was used as internal control. **B.** Graph showing fold-change values of SCARA5 protein expression in cells transfected with siRNA to SCARA5 versus cells transfected with Scrambled siRNA, normalized on vinculin levels.

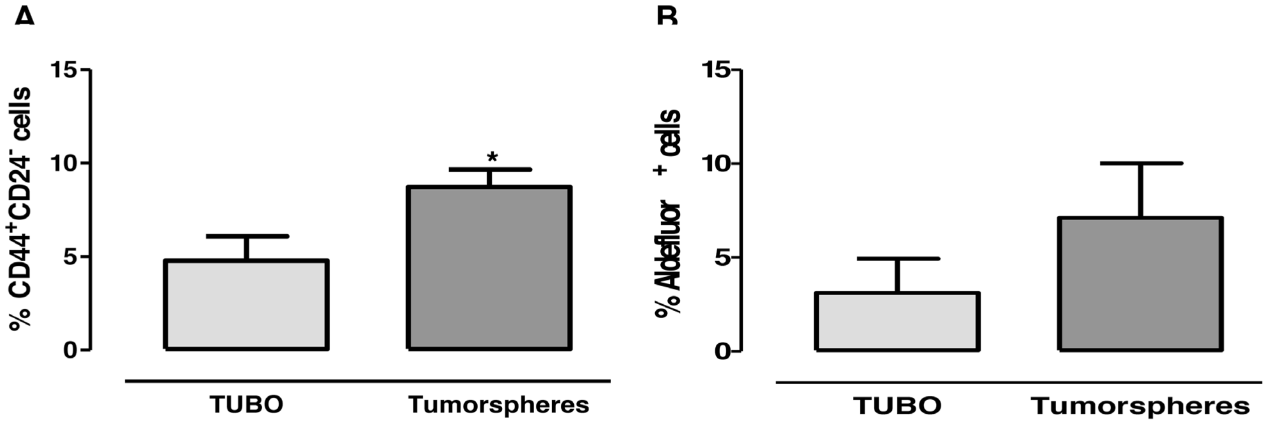

Supplementary Figure S2: Tumorsphere-induced tumors maintan a higher CSC percentage than TUBO tumors *in vivo*. FACS analysis of **A.** $CD44^{+}CD24^{-}$ and **B.** $Aldefluor^{+}$ CSC in 10 mm mean diameter tumors generated by s.c. injection of 10^{5} TUBO or 10^{4} tumosphere-derived cells in BALB/c mice. The graphs show the mean ± SEM of $CD44^{+}CD24^{-}$ or $Aldefluor^{+}$ cells from 7 tumors per group.*, P<0.05, Student's t test.

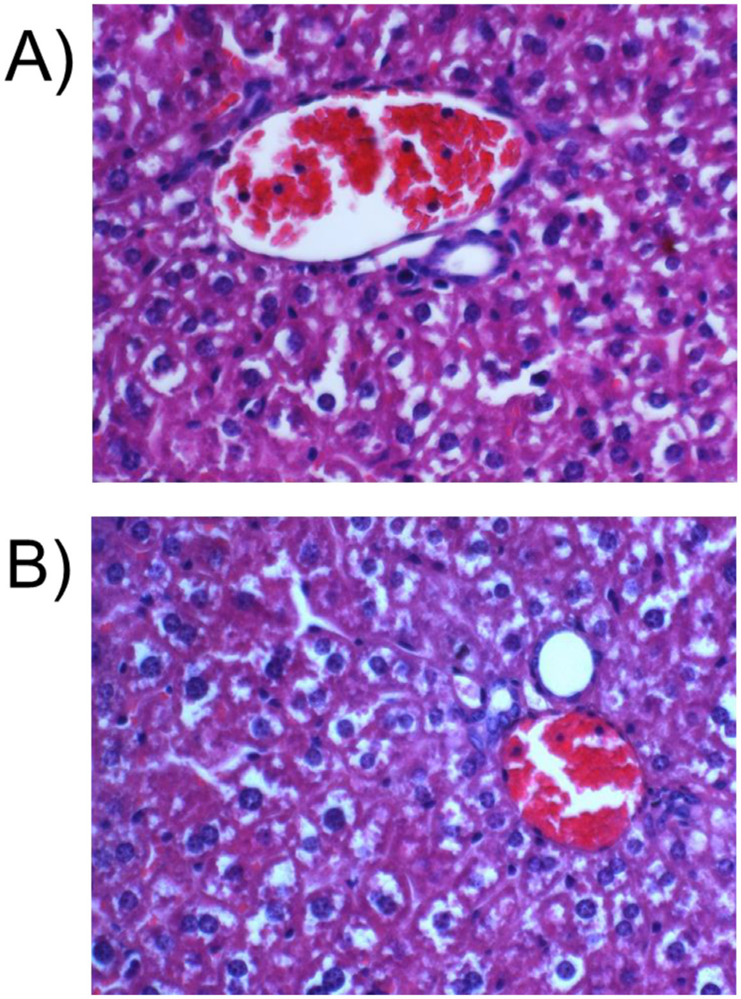

Supplementary Figure S3: APO-Curcumin treatment does not cause liver damage. Representative histological liver pictures from APO-curcumin **A.** and Apoferritin **B.** treated mice. 5µm tissue slices were stained with hematoxylin and eosin.

Supplementary Table S1: T_2 weighted MRI image Signal Intensities (S.I.)

	S.I. TUBO differentiated	*S.I.* TUBO tumorspheres	*S.I.* MDA-MB-231 differentiated	*S.I.* MDA-MB-231 tumorspheres
CTRL	$2.38 \times 10^5 \pm 0.20$	$2.39 \times 10^5 \pm 0.25$	$2.27 \times 10^5 \pm 0.19$	$2.21 \times 10^5 \pm 0.063$
Ferritin	$2.38 \times 10^5 \pm 0.17$	$0.186 \times 10^5 \pm 0.051$	$1.72 \times 10^5 \pm 0.059$	$0.21 \times 10^5 \pm 0.070$

Signal Intensity (S.I.) measured on T_2-weighted MRI images of TUBO and MDA-MB-231 cells (differentiated and tumorspheres) reported in Figure 2C.

Supplementary Table S2: Longitudinal Relaxation Rates (R_{1obs}, s^{-1}) measured at 7T

	$R_{1obs}(s^{-1})$ TUBO differentiated	$R_{1obs}(s^{-1})$ TUBO tumorspheres	$R_{1obs}(s^{-1})$ MDA-MB-231 differentiated	$R_{1obs}(s^{-1})$ MDA-MB-231 tumorspheres
CTRL	0.436±0.015	0.449±0.006	0.384±0.017	0.429±0.030
Apo-Gd	0.52±0.0068	0.597±0.008	0.483±0.015	0.717±0.020

Longitudinal Relaxation Rates (s^{-1}) measured on T_1-weighted MRI images of TUBO and MDA-MB-231 cells (differentiated and tumorspheres) reported in Figure 2C.
